# Tissue Response to Neural Implants: The Use of Model Systems Toward New Design Solutions of Implantable Microelectrodes

**DOI:** 10.3389/fnins.2019.00689

**Published:** 2019-07-05

**Authors:** Maurizio Gulino, Donghoon Kim, Salvador Pané, Sofia Duque Santos, Ana Paula Pêgo

**Affiliations:** ^1^i3S – Instituto de Investigação e Inovação em Saúde, Universidade do Porto, Porto, Portugal; ^2^INEB – Instituto de Engenharia Biomédica, Universidade do Porto, Porto, Portugal; ^3^FEUP – Faculdade de Engenharia, Universidade do Porto, Porto, Portugal; ^4^Multi-Scale Robotics Lab (MSRL), Institute of Robotics and Intelligent Systems (IRIS), ETH Zurich, Zurich, Switzerland; ^5^ICBAS – Instituto de Ciências Biomédicas Abel Salazar, Universidade do Porto, Porto, Portugal

**Keywords:** neural tissue response, microelectrodes, foreign body reaction, brain slice cultures, neural tissue engineering, deep brain stimulation

## Abstract

The development of implantable neuroelectrodes is advancing rapidly as these tools are becoming increasingly ubiquitous in clinical practice, especially for the treatment of traumatic and neurodegenerative disorders. Electrodes have been exploited in a wide number of neural interface devices, such as deep brain stimulation, which is one of the most successful therapies with proven efficacy in the treatment of diseases like Parkinson or epilepsy. However, one of the main caveats related to the clinical application of electrodes is the nervous tissue response at the injury site, characterized by a cascade of inflammatory events, which culminate in chronic inflammation, and, in turn, result in the failure of the implant over extended periods of time. To overcome current limitations of the most widespread macroelectrode based systems, new design strategies and the development of innovative materials with superior biocompatibility characteristics are currently being investigated. This review describes the current state of the art of *in vitro, ex vivo*, and *in vivo* models available for the study of neural tissue response to implantable microelectrodes. We particularly highlight new models with increased complexity that closely mimic *in vivo* scenarios and that can serve as promising alternatives to animal studies for investigation of microelectrodes in neural tissues. Additionally, we also express our view on the impact of the progress in the field of neural tissue engineering on neural implant research.

## Implantable Electrodes in Neurological and Neuropsychiatric Disorders

### Clinical Applications

Recent technological progress in the field of brain-machine interfaces boosted the development of innovative tools and electrodes for neurophysiological research and neurostimulation applications to treat neuro disability-related conditions.

Deep brain stimulation (DBS) is an invasive neurosurgical operation consisting in the delivery of electrical impulses to specific areas of the brain by means of implantable electrodes connected to a pulse generator. The concept of DBS is the generation of action potentials resulting in beneficial neurochemical effects, such as the recovery of disrupted neural circuits and physiological brain function. For specific applications, DBS is a U. S. “Food and Drug Administration” (FDA) approved technique which is already applied in the clinic for a vast number of neurological dysfunctions (Coenen et al., 2015; Revell, 2015). In patients affected by Parkinson’s disease, DBS at the level of the globus pallidus or subthalamic nucleus is able to reduce bradykinesia, dystonia, as well as walking problems, allowing for substantial improvements in the quality of life (Ramirez-Zamora and Ostrem, 2018). In patients affected by dystonia, globus pallidus DBS has been shown to reduce tremors and involuntary motor contraction, with persistent effects after several years (Meoni et al., 2017). For essential tremors, the principal targets for DBS to reduce tremors with lower stimulation amplitudes and fewer side effects than previous treatments are the posterior subthalamic area and the zona incerta (Holslag et al., 2017; Barbe et al., 2018). In patients with medically refractory epilepsy, long-term DBS at the level of the anterior nucleus of the thalamus is an U. S. FDA-approved therapy that is able to reduce epileptic episodes and ensure perdurable improvements in the quality of life for years (Salanova, 2018). For the treatment of chronic and neuropathic pain, DBS is a supported therapy in Europe, although not currently approved by the U. S. FDA. Several clinical studies report a reduction of pain in patients with amputations, post-stroke pain, cranial and facial pain (Farrell et al., 2018). DBS was also found to be a valid therapeutic approach for the treatment of psychiatric disorders. Ventral capsule/ventral striatum DBS is another U. S. FDA-approved treatment under a humanitarian device exemption for patients affected by obsessive-compulsive disorder (Karas et al., 2019). A relevant efficacy of DBS has been also observed for the treatment of refractory depression. Clinical application of DBS in patients non-responsive to anti-depressant treatments reported a remission of depression after chronic stimulation of various brain targets, with a decrease in negativity and sadness, reduction of cerebral blood flow at the level of the limbic-cortical circuitry, and improvements in memory and motor function (Dandekar et al., 2018; Drobisz and Damborská, 2019).

Spinal cord stimulation (SCS) has shown to be an effective strategy for the treatment of different diseases. SCS is being successfully used to treat angina pectoris pain, low back, and leg pain and peripheral limb ischemia (Song et al., 2014). Clinical studies reported beneficial effects of SCS combined with activity-based training in the recovery of motor function and muscle activation patterns in patients that suffered spinal cord injury (Rejc et al., 2017).

Besides the applications described above, the use of implantable electrodes for neurostimulation therapy is continuing to expand toward many other medical conditions. DBS has been recently proposed for the treatment of pain, dystonia and motor symptoms in post-stroke, although additional investigations are necessary to identify specific brain districts to improve the effectiveness of the treatment (Elias et al., 2018). In a study involving patients affected by Tourette syndrome, DBS of the anterior and posterior globus pallidus, centromedian thalamus and anterior limb of internal capsule showed common positive results after 1 year of treatment in the reduction of motor tic symptoms (Martinez-Ramirez et al., 2018). Partial improvements have been described in clinical trials involving patients affected by Alzheimer’s disease, with positive effects in cognition, reversal of memory and reduction of altered glucose metabolism (Mao et al., 2018).

As seen above, the progress in neurostimulation technology and the increased knowledge of the neurophysiology of the central nervous system (CNS) opened the way to new therapeutic approaches for the treatment of a vast number of neurological disorders and neuropsychiatric conditions. Although well established and approved for some diseases, additional trials and experimental work need to be conducted to better define the ideal brain targets, stimulation variables, and electrode design in order to ameliorate the clinical outcomes. Due to the impact of the inflammatory response and tissue encapsulation elicited by traditional DBS macroelectrodes, the field of neuroengineering is progressing toward the employment of implantable microelectrodes (Daniele and Bragato, 2014). Traditional DBS electrodes present several drawbacks such as the rigidity of the materials employed for fabrication as well as a high size which exacerbate the neuroinflammatory response and tissue damage. Although they have a higher activation radius compared to microelectrodes, macroelectrode implantation is often associated to a wrong positioning in the interested area causing a decrease in therapeutic efficacy of DBS (Kloc et al., 2017; Morishita et al., 2017). In the case of small target areas, the reduced dimensions of microelectrodes can provide a better targeting accuracy ensuring an increased therapeutic efficacy of DBS and tissue integration for chronic applications (Desai et al., 2012; Du et al., 2017; Ganji et al., 2017). These miniaturized devices offer additional advantages such as reduced tissue damage and impedance, increased signal-to-noise ratio and neuronal activation compared to traditional electrodes (Desai et al., 2014). A key component contributing to microelectrode design and, ultimately, to clinical performance is the selection of the materials for the device. Such materials should not only satisfy the mechanical and the structural requirements for the efficient electrochemical performance but also provide a durable and biocompatible interface with the brain tissue. In the following section, the current materials used in microelectrode fabrication are presented.

### Current Materials for Microelectrode Fabrication

For the long-lasting efficient microelectrodes, the material should not only function properly *in vivo* but also be biocompatible and durable for the protection of both the patient and the device (Szostak et al., 2017). As different materials show dissimilar behavior in the tissue environment the choice of the implant material is crucial. To mitigate foreign body reactions and corrosion/degradation of the structures, electrical, chemical, and mechanical properties of materials, such as chemical composition, crystallinity, surface morphology, the electrode microstructure, and Young’s modulus (a measurement of elasticity) need to be carefully considered (Cogan, 2008; Williams, 2008, 2009). With the advance of the micro-fabrication techniques, silicon and polymers have been widely employed as the substrate materials, while metals, carbon nanotubes, conductive polymers as electrode site materials (Samba et al., 2015; Yi et al., 2015; Antensteiner and Abidian, 2017).

Typical microelectrodes are designed to have either an array of microwires or micro-electromechanical system (MEMS) arrays. Microwires are generally composed of metals such as gold, tungsten, and stainless-steel, coated with insulators. Two different types of silicon substrate-based MEMS micro-machined electrodes, i.e., the Utah array (Normann and Fernandez, 2016; Wendelken et al., 2017) and the Michigan array (Kipke et al., 2008; Kiss et al., 2015), have been significantly exploited for decades. However, a huge mechanical mismatch between hard metals/silicon (E ∼ 10 to 100 GPa) and soft brain tissue (0.4–15 kPa) results in substantial strain at the tissue-electrode interface, causing local physical damage that result in inflammation and neural degeneration (Polikov et al., 2005; Harris et al., 2011; Merrill, 2014). The inflammatory process may hinder the stimulation of neuronal cells, as well as it may contribute to device failure as a result of electrode degradation (Kozai et al., 2015a; McCreery et al., 2016). However, it must be highlighted that not all the microelectrode types exhibit the same degradation profile or that there is a direct correlation between electrode failure and the acute inflammatory response (Gaire et al., 2018a). Several efforts have been reported to overcome the drawbacks from the mechanical mismatch between electrode and tissue by implementing materials with lower Young’s modulus, e.g., flexible and biocompatible polymer substrates (Trevathan et al., 2019). Polyimide- (Lai et al., 2012) and parylene-based MEMS (Hess et al., 2011) electrodes have been heavily investigated for their improved mechanical properties, easy access to fabricate, and capability to introduce bioactive molecules at the interface to facilitate long-term interaction with the tissue. As chronic stimulation electrode site materials, metals including tungsten, platinum, iridium, tantalum pentoxide, and titanium nitride have been extensively used for their electrical charge-injection properties and biocompatibility (Cogan, 2008; Fattahi et al., 2014; Meijs et al., 2015). For its remarkably increased charge storage and injection capacity and high corrosion resistivity, iridium oxide has been also widely utilized as a coating material (Meyer et al., 2001; Hasenkamp et al., 2009) to enhance the performance and the durability of the electrode. Carbon nanotubes (Jiang et al., 2011; Schmidt et al., 2013) and conducting polymers such as poly(3,4-ethylene dioxythiophene) (PEDOT) and poly(styrene sulfonate) (PSS) (Cui and Martin, 2003; Pranti et al., 2018) are attracting considerable attention as alternatives to the metal electrodes and coatings for their biocompatibility and tunable electrical properties.

An important challenge in microelectrode fabrication for neural stimulation is the identification of smart materials that are able to provide enhanced biocompatibility. So far, all the current microelectrodes are recognized, in the long run, as foreign bodies by the nervous tissue. As the difference in Young’s modulus between the electrode and tissue is the main factor that causes damage and inflammation, most research is focused on using materials with low Young’s modulus for both substrates and electrode sites. However, it should be also taken into account that mechanical strain from the motion artifacts, such as bending of conducting material, can cause changes in resistance or capacitance of materials. As a result, this can affect the electrical signals of the electrode and can result in unintended performance (Michelson et al., 2019). Thus, the balance between Young’s modulus and the electrical properties need to be carefully considered in designing microelectrodes for neural stimulation.

## Foreign Body Response as a Cause of Implant Failure

The nervous tissue response to implantable microelectrodes is a complex process characterized by a cascade of biochemical alterations and chemical reactions occurring at the level of the tissue-material interface. These biochemical and chemical alterations may culminate in an undesired foreign body response. Additionally, changes in the inherent properties of the electrode after long-term implantation, for example due to corrosion, may further impair its tissue compatibility and durability. Body fluids and tissues are highly corrosive environments characterized by an elevated presence of oxygen, saline electrolytes, macromolecules and dissolved ions that can cause the electrochemical detachment of microelectrode surface. Once surgically implanted, microelectrodes must remain intact for several years to ensure the efficacy of the therapy and device functionality. To provide successful integration, reliability, and durability once implanted in the brain tissue, microelectrodes must fulfill the following requirements:

• Biocompatibility: the surface of the microelectrode must be non-toxic for neural cells without causing any adverse effects to the surrounding tissues.
• Biomimicry: the surface has to mimic the physicochemical and mechanical characteristics of the extracellular matrix (ECM) in order to promote neurite outgrowth toward the electrode surface and to avoid activation and recruitment of glial cells and fibroblasts that can contribute to the encapsulation of the electrodes.
• Biostability: microelectrodes need to maintain their physical integrity, electrochemical stability, and functionality, and resist the highly corrosive tissue microenvironment without undergoing any structural modification.

Microelectrode implantation causes unavoidable damage to the tissue, triggering a series of neuroinflammatory reactions, which are part of the natural wound healing process that can seriously affect the stimulating site integrity and hamper the electrochemical performance in long-term implantations (Salatino et al., 2018). The complexity of such a process can be described by dividing it into two coupled factors: biotic factors, represented by the effects of cells and tissue reactions occurring at the surface; and the abiotic factors, related to the characteristic of the material itself.

Biotic factors include the blood-brain barrier (BBB) rupture, protein absorption at the material surface, immune cells and fibroblast recruitment, increased production of radical ions, cell death and the formation of the insulating glial scar around the electrode surface, which hampers blood supply and ionic equilibrium at the injury site. The abiotic factors are represented by the physicochemical surface modifications such as the dissolution of passive films, material-related impedance, the failure of the stimulating site integrity, the formation of electrochemical cells at the level of the surface that can evolve in crevices or pits. Biotic and abiotic factors cannot be considered as two separated processes, as they are strictly dependent and occur simultaneously interacting in different manners during the lifetime of the electrode. The complexity of such a process is not totally understood and further research is needed to clarify whether the contributions of these interrelated factors occur and what are the most effective intervention strategies.

In this section, we will provide a description of the main cascade of biochemical and cellular events occurring upon brain microelectrode implantation with a focus on the biotic reactions.

**FIGURE 1:**
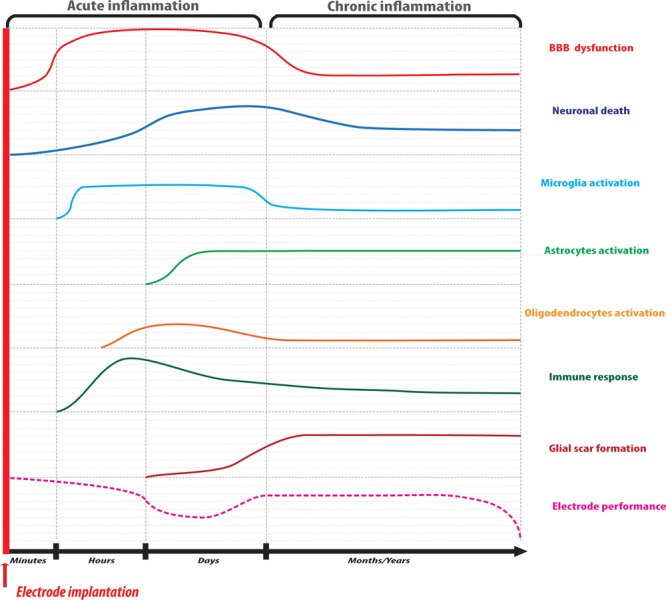
Schematic timeline representation of the reactions involved in the process of neural tissue response to implantable microelectrodes. The acute phase of inflammation is characterized by BBB disruption and neuronal death due to mechanical insult followed by glial activation and immune cell recruitment at the injury site. Microelectrode performance may be hampered at this level due to mechanical mismatch with the tissue accompanied by a temporary recovery. In the chronic phase of inflammation, a glial fibrotic scar surrounds the microelectrode impeding material and stimulating site integrity that, ultimately, may result in implant failure.

The process that leads to glial scar formation due to implantation and can culminate in the encapsulation of an implant can be divided into two phases (Figure 1). An acute phase that starts immediately after device implantation and characterized by BBB dysfunction and glial cell activation, followed by a chronic phase characterized by an immune response and the development of a glial scar around the implant.

The first and one of the most critical events occurring during device implantation is the rupture of the BBB. The implantation causes a break at the level of the endothelial vessels, with a reduction of blood flow and oxygen supply, accumulation of plasma proteins and pro-inflammatory factors, and myeloid cell infiltration (Kozai et al., 2015b). Cell membrane damage by mechanical stress causes an increase in Ca^2+^ concentration either by its release through the pores in the cell membrane and by disturbances in the electrochemical potential of Na^+^ channels, which lead to membrane depolarization (Eles et al., 2018; Salatino et al., 2019). Membrane depolarization, in turn, leads to the increase in intracellular Ca^2+^, neurotransmitters release from presynaptic terminations (Eles et al., 2018), resulting in excessive production of reactive oxygen species (ROS) due to mitochondrial damage (Ereifej et al., 2018). The BBB breach has been shown to be crucial in the triggering of biochemical pathways responsible for neuronal degeneration and glial activation (Saxena et al., 2013). Some plasma proteins such as globulins, fibrinogen, thrombin, plasmin, and albumin can accumulate at the injury site through the BBB gap and can be adsorbed at the electrode surface.

### Microglia

Glial activation represents the main cellular event involved in the neuroinflammatory response. As the resident macrophage cells of the brain, microglia are ubiquitous in the CNS, and they become activated to carry out their neuroprotective functions immediately after electrode implantation. Once activated, they act as principal effectors of the neuroinflammatory response and can orchestrate the process through cross-talk with astrocytes and oligodendrocytes. It is already been accepted that microglia exist in “pro-inflammatory” and “anti-inflammatory” phenotypes. The former is the “classical activation” phenotype, in which cells secrets pro-inflammatory cytokines and contribute to neuronal injury; in the case of the latter phenotype cells secret anti-inflammatory cytokines and contribute to tissue remodeling and repair, phagocytosis of cell debris, as well as antagonize pro-inflammatory activity. In the early hours post-implantation, pro-inflammatory microglia secrete pro-inflammatory cytokines and chemokines such as interleukins IL-1α, IL-1β, IL-6, tumor necrosis factor (TNF-α), monocyte chemoattractant protein 1 (MCP-1) (Sawyer et al., 2014), ROS and reactive nitrogen species (RNS), determining massive immune cell recruitment and additional cytokine production (Hermann et al., 2018a). The lack of oxygen redox homeostasis acts directly on microglia, astroglia and endothelial cells causing activation of metalloproteinase, downregulation of tight junctions and adherens junction genes in the first hours after BBB injury (Bennett et al., 2018), facilitating the entrance of infiltrating macrophages, which will also have a crucial role in neurodegeneration (Ravikumar et al., 2014).

### Astrocytes

Astrocytes are another type of neuroglia that is very affected after implantation. Astrocytes perform many functions, including biochemical support of endothelial cells that form the BBB, supplying of nutrients to the nervous tissue, maintenance of extracellular ion balance, having a key role in the repair and scarring process of the brain. In analogy to microglia, astrocytes exist in a pro-inflammatory phenotype and an anti-inflammatory phenotype. Pro-inflammatory astrocytes are activated by pro-inflammatory microglia and secret neurotoxins creating a hostile environment for neuronal and oligodendrocytes regeneration. Pro-inflammatory astrocytes are activated by Il-1β, TNF-α and complement component 1q (C1q) from microglia, responding immediately to electrode implantation and changes in neuronal activity, accumulating in the vicinity of the microelectrode during the first week after implantation (Liddelow et al., 2017). At this level, astrocytes alter the neuronal viability causing neuronal loss, reduction of fiber density and overexpression of glial fibrillary protein (GFAP) and vimentin, which are critical for their change in morphology and extension of the protrusions at the injury site (Woolley et al., 2013; Moeendarbary et al., 2017).

### Neurons

The neuronal loss also occurs immediately after implantation. The mechanical stress caused by electrode entry into the tissue leads to axonal morphological changes, neuronal membrane disruption with the formation of axonal blebs as an indication of neuronal damage. Oxidative stress in neurons is caused by the increase in intracellular calcium through glutamate-N-methyl-D-aspartate receptor (NMDA) activation, as well as by ROS and RNS produced by microglia and astrocytes, causing mitochondrial dysfunction. Neuronal degeneration and neuroinflammation are exacerbated by the persistent secretion of proinflammatory cytokines and glial fibrillary proteins deposition by microglia and astrocytes, finally forming the glial scar (Figure 1). As a consequence of this neuronal death at the tissue-electrode interface, the distance between electrode and synapses grows in time, hampering electrical stimulation performance.

### Oligodendrocytes

Oligodendrocyte cell death will also occur at the implantation site. Either due to cell membrane damage or as a result of neuronal cell death or axonal degeneration. Oligodendrocytes play the important function of ensuring axonal support and myelin production and maintenance. In the acute phase of foreign body reaction, oligodendrocytes become highly sensitive to oxidative stress by ROS and RNS as well as excitotoxic damage by glutamate oversignaling. Apoptosis of oligodendrocytes leads to demyelination and can also culminate in neuronal death depending on the extent of the event. In the adult brain, one can find not only myelinating oligodendrocytes but also cells in the form of neuron-glia antigen 2-expressing glial cells (NG2) precursors, which are present from development to the adult phase, denominated oligodendrocyte progenitor cells. *In vivo* studies showed that new NG2 precursors become activated by proinflammatory factors secreted by reactive microglia and can be seen migrating to the injury site 12 h post-implantation (Wellman and Kozai, 2018). But there, NG2 precursors preferentially differentiate to astrocytes and further move toward the implant participating in the formation of the glial scar, not contributing to the turnover of new myelinating oligodendrocytes (Wellman et al., 2018a).

### The Glial Scar

Over 2 weeks post-implantation, in the chronic phase of the process, it has been observed that astrocytes and microglia have their protrusions extended toward the material surface creating a non-permeable barrier between the implant and the tissue over 2 weeks post-implantation (Wellman and Kozai, 2017). At this stage, fibroblasts have reached the inflammation core from meninges and secret ECM proteins such as fibronectin, type IV collagen, laminin, and chondroitin sulfate proteoglycans, also contributing to the formation of the glial scar and the encapsulation of the microelectrode at the parenchymal level (Dias and Göritz, 2018). This insulating barrier constitutes a hostile environment that hampers electrophysiological performance due to the absence of contact between microelectrode and neurons, leading to the failure of the implant over extended periods of time (Figure 1).

Despite all the efforts that have been carried out to study the dynamics of glial scar in injury and disease, additional investigations are required to understand the specificities of the foreign body response in the context of electrode implantation, to uncover the most effective intervention strategies to promote microelectrode integration in the CNS (Salatino et al., 2018). Besides, it is important to take in consideration that other aspects can influence glial scar heterogeneity, such as the type of microelectrode material used, the cerebral anatomical district of implantation, as well as the pathological context in which it is applied. Hence, the use of modeling systems that can mimic specific *in vivo* pathological conditions is a great opportunity to move toward the establishment of innovative approaches on which to base future microelectrode design.

## Experimental Models to Study Foreign Body Response to Neural Implants

**FIGURE 2:**
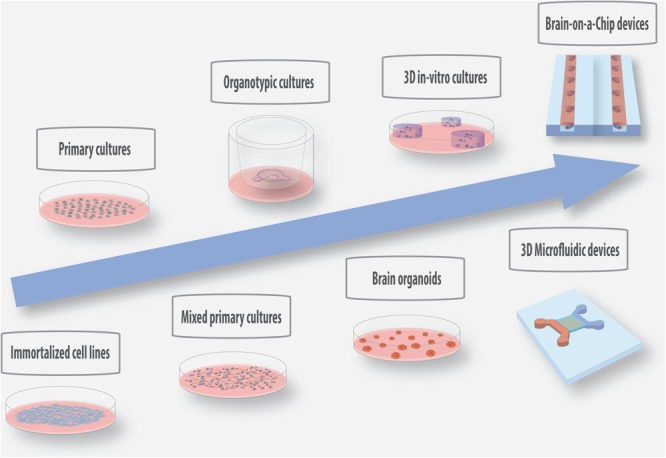
Schematic representation of the current and promising *in vitro*/*ex vivo* models with increased physiological relevance for the screening of materials and coatings for the development of implantable microelectrodes.

Despite the great advances achieved in neural interface technology, some questions related to the molecular and cellular events involved in nervous tissue response to implantable microelectrodes remain unanswered. Several *in vivo* studies have been performed to identify critical aspects and design solutions to inhibit glial encapsulation in chronic applications. However, due to their cost, time consumption and complexity *in vivo* models are not ideal systems to investigate the detailed cues of tissue-electrode interactions. With this purpose, substantial research has been focused on the development of relevant *in vitro* biological platforms of increased complexity to test new materials and biosurfaces, which can offer a controlled and reproducible platform for high-throughput screenings. In the following paragraphs, we provide an overview of the current *in vitro*/*ex vivo*/*in vivo* models employed in microelectrode research, as well as a description of new promising 3D *in vitro* technologies with increased complexity that can be of added value to future investigations in this field (Figure 2).

### *In vitro* Models

One of the main goals in the design and testing of new materials as well as coatings for microelectrodes is to reduce glial cell activation while allowing/inducing neuronal synaptic activity. *In vitro* 2D cultures represent the simplest model to investigate the impact of materials properties on the cellular response (Table 1).

**Table 1:** Current and novel models explored for the study of biocompatibility of materials for neural applications and assessment of electrochemical performance and durability of implantable microelectrodes.

Model	Specifics	Purpose of the study	References
Immortalized cell cultures	BV-2 mouse microglia cell line	Cellular responses to nanotextured silicon surfaces	Persheyev et al., 2011
	C6 rat astrocytoma cell line	Effect of nanopatterned poly(methyl methacrylate) surfaces on astrocyte reactivity	Ereifej et al., 2013b
	PC-12 rat pheochromocytoma cell line	Biocompatibility of polyurethane/poly(vinyl alcohol) hydrogel coatings	Li et al., 2015
	SH-SY5Y human neuroblastoma cell line	Cytotoxicity of nanostructured Pt-coatings	Boehler et al., 2015
	NIH/3T3 mouse embryonic fibroblast cell line	Cytocompatibility of polyaniline surfaces	Rejmontová et al., 2016
	L929 mouse fibroblast cell line	Cytotoxicity of poly(3,4-ethylenedioxythiophene): glycosaminoglycan (PEDOT:GAG)	Mantione et al., 2016
	NIH/3T3 mouse embryonic fibroblast cell line	Biocompatibility of hydrophilic copolymers	Hadler et al., 2017
Primary cell cultures	Primary microglia	Cellular responses to nanostructured titanium oxide surfaces	De Astis et al., 2013
	Primary rat microglia	Response of microglia to P(TMC-CL)	Pires et al., 2015
	Primary mouse hippocampal neurons	Effects of nanotopography on neuronal cell signaling	Onesto et al., 2017
	Primary rat hippocampal neurons	Biocompatibility of poly(3,4-ethylenedioxythiophene) doped with poly(styrene sulfonate) MEAs	Koutsouras et al., 2017
	Primary human dermal fibroblasts	Study of surface nano-topography and chemistry on collagen I and III production	Bachhuka et al., 2017
	Primary rat cortical neurons	Development of a MEA-based *in vitro* model for drug screening	Bradley et al., 2018
	Primary rat cortical and spinal cord Astrocytes	Response of astrocytes to fiber surface nanotopography	Johnson et al., 2018
2D mixed cell cultures	Primary rat mixed microglia, astrocytes and oligodendrocytes	2D *in vitro* glial scar assay to test biocompatibility of insulating silicone polymer coatings	Achyuta et al., 2010
	Primary rat astrocytes derived from neurospheres and rat embryonic spinal cord cells	2D *in vitro* model of spinal cord injury for drug screening	Boomkamp et al., 2012
	Primary rat mixed neurons, microglia, astrocytes and oligodendrocytes	2D *in vitro* glial scar assay to test cellular responses of dip-coated PEG films	Sommakia et al., 2014
	Rat primary astrocytes and dorsal root ganglia neurons	2D *in vitro* model of spinal cord injury to study isotropic-to-anisotropic cellular transitions	Zuidema et al., 2015
	Primary cortical neurons and astrocytes	Cellular responses to nanoporous gold surfaces	Chapman et al., 2016
3D *in vitro* cultures	Primary rat microglia and astrocytes	Hyaluronic-based hydrogel as a 3D model to test electrode biocompatibility	Jeffery et al., 2014
	Primary mouse mixed neurons, microglia, astrocytes and oligodendrocytes	Alvetex membrane scaffold as a 3D culture for high-throughput screening	Smith et al., 2015
	Primary rat cortical neurons and astrocytes	Alginate-based hydrogel as a 3D model of glial scar	Rocha et al., 2015
	Primary rat mixed neurons, microglia, astrocytes and oligodendrocytes	Type I collagen-based hydrogel as a 3D model of glial scar	Spencer et al., 2017a
	Primary rat mixed microglia, astrocytes and oligodendrocytes	Hyaluronic acid-based hydrogel as a 3D model of glial scar	Koss et al., 2017
	PC-12, C6, human iPSC and rat primary dorsal root ganglia neurons	Type I collagen-based 3D hydrogel for high-throughput study of neurodegeneration	O’Rourke et al., 2017
Organotypic cultures	Rat organotypic hippocampal slices	Biocompatibility of silicon-based electrode arrays	Kristensen et al., 2001
	Rat organotypic brain slices	Biocompatibility of nanopatterned polydimethylsiloxane	Ereifej et al., 2013a
	Mouse organotypic spinal cord slices	Characterization of the ability of 3D meshed-carbon nanotubes to support neurite regrowth	Usmani et al., 2016
	Chicken embryo organotypic brain and liver slices	Cyto-biocompatibility of thin-film transistors	Leclerc et al., 2017
Brain organoids	Human induced pluripotent stem cells (hiPSC) derived organoids	Model of autosomal recessive primary microcephaly	Gabriel and Gopalakrishnan, 2017
	Human embryonic stem cells (hESCs) derived organoids	Testing functionalized borosilicate glass capillaries for glutamate detection	Nasr et al., 2018
	Human primary microvascular endothelial cells, perycites, and astrocytes, mixed iPSC derived oligodendrocytes, microglia and neural stem cells	3D spheroid model of BBB for High-Throughput neurotoxicity screening and disease modeling	Nzou et al., 2018
Microfluidics	hiPSC-derived neurons and astrocytes	High-throughput screening of neurotoxic compounds	Wevers et al., 2016
	Ventral spinal cord motoneurons, rat primary meningeal fibroblasts and astrocytes	*In vitro* model of glial scar	Li et al., 2017
	Pre-differentiated hiPSC lines derived from skin fibroblasts	Brain organoids on chip for the study of impaired neurogenesis induced by cadmium	Yin et al., 2018
*In vivo* models	Unilateral 6-OHDA injection in adult rats to model nigrostriatal degeneration of Parkinson’s disease	Analysis of c-fos expression after DBS of the pedunculopontine tegmental nucleus	Saryyeva et al., 2011
	Unilateral 6-OHDA injection Adult rats to model nigrostriatal degeneration of Parkinson’s disease	Analysis of subthalamic nucleus-DBS on behavioral performance	Badstuebner et al., 2017
	Rat model of retinitis pigmentosa	Analysis of a fully organic retinal prosthesis to treat degenerative blindness	Maya-vetencourt et al., 2017
	Induction of status epilepticus through injection of pilocarpine in adult rats	Study of long-term DBS of the anterior thalamic nucleus	Ferreira et al., 2018

*BV-2, mouse microglia cell line (CVCL_0182); C6, rat astrocytoma cell line (CVCL_0194); DBS, deep brain stimulation; C-fos, transcription factor subunit (OMIM: 164810); hESCs, human embryonic stem cells; hiPSC, human induced pluripotent stem cells; L929, mouse fibroblast cell line (CVCL_AR58); MEA, multi-electrode array; NIH/3T3, mouse embryonic fibroblast cell line (CVCL_0594); 6-OHDA, 6-hydroxidopamine (5-(2-aminoethyl)benzene-1,2,4-triol); PEDOT:GAG, poly(3,4-ethylenedioxythiophene): glycosaminoglycan; PEG, poly(ethylene glycol); PC-12, rat pheochromocytoma cell line (CVCL_F659); P(TMC-CL), poly(trimethylene carbonate-co-epsilon-caprolactone); SH-SY5Y, human neuroblastoma cell line (CVCL_0019).*

#### Immortalized Cell Line Cultures

The use of relevant immortalized cell lines can provide significant insights regarding material biocompatibility and can contribute to the study of cell-microelectrode material interactions. The experimental conditions are controlled in terms of cell identity, adaptability, and reproducibility. Immortalized cell lines are simple to culture, can be grown for indefinite periods of time, maintaining genotypic stability and allowing the readily generation of large amounts of cells for analysis. Fibroblasts are one of the most well-characterized cell types to study the biocompatibility and the cell-adhesion properties of metals and coatings for biomedical devices. These cells play a critical role in the formation of the fibrotic scar in the late phases of inflammation. The modulation of their adhesion and interactions with the implants is crucial for ensuring a stable device performance. Fibroblast cell lines such as L929 and NIH/3T3 have been widely used to conduct standard material biocompatibility and cytotoxicity testing in accordance with the International Organization for Standardization (ISO) norm 10993-5. The latter defines a series of test methods employing cell monolayers in contact with the material or with material extracts to assess toxicity. In particular, the mouse embryonic NIH/3T3 fibroblasts are a well-characterized cell type used by FDA for biocompatibility testing of materials and coatings for neural devices. Namely, these were employed to assess surface properties of various preparations of polymeric conductive materials for neural devices applications (Mantione et al., 2016; Rejmontová et al., 2016; Hadler et al., 2017; Morin and He, 2017;Wang J. et al., 2018).

Given their central role in orchestrating nervous tissue response to microelectrodes, glial cell were also employed to investigate the effects of new surfaces and designs on cell adhesion, morphology and activation (Persheyev et al., 2011; Ereifej et al., 2013b; Lee et al., 2014). Bérces et al. (2018) employed the immortalized murine microglial cell line BV-2 to investigate the effects of nanotopography on silica and platinum surfaces and compared their behavior with neural stem cells. They showed that while BV-2 cells grew indifferently on nanostructured and non-coated samples, neural stem cells grown on nanostructured surfaces displayed a decrease in cell viability, adhesion and a tendency to adhere to each other instead of to the surface. C6 glioma cells were employed to investigate the biocompatibility of Pt-grown carbon nanofibers coatings for enzymatic glutamate biosensors and compared to Ni-grown nanofibers, showing that cells exhibit different cell adhesion and morphology at different dimensions of nanofibers (Isoaho et al., 2018). Several studies have been conducted to investigate the biocompatibility and the effect of surface properties, like roughness and topography, on neuronal cells. Rat pheochromocytoma PC12 neuronal cell lines are the most used to study the ability of new biomaterials to promote neuronal adhesion and neurite outgrowth (Klymov et al., 2015; Li et al., 2015). Wandiyanto et al. (2018) have recently shown that PC12 cells grown on anti-bactericidal titanium nanostructures displayed enhanced proliferation, differentiation and neurite outgrowth compared to non-nanostructured surfaces. Tasnim et al. (2018) recently investigated, using the SH-SY5Y cell line, the biocompatibility of graphene oxide coating for commercially available 316 stainless steel. They showed that graphene oxide coating enables cell adhesion, proliferation and viability, as well as reduces ROS production compared to bare 316 stainless steel. Nissan et al. (2017) also employed SH-SY5Y to investigate the nanotopographical effects of silver nanoline coatings, showing that cells positively respond by increasing neurite outgrowth and branching points compared to unmodified silica wafers.

While immortalized cell lines are a versatile and readily available tool in the early phases of material testing (Table 1), these do not have the same biological relevance and response of their primary counterparts or even. Immortalized cell lines display evident phenotypic and physiological differences from the cell type of origin. These differences can be due to the cell source (many times tumor samples), immortalization process, the propagation and differentiation protocols, as well as the culture conditions and medium composition (Kaur and Dufour, 2012; Lorsch et al., 2014). Consequently, despite being from a similar cell type, immortalized cells can display different viability, metabolic and adhesive properties, as well as different expression profiles and cytotoxic responses to materials. In the context of neurophysiological investigations, they can display different electrophysiological responses to stimulations/recordings, thus they are not the best candidates to serve as models to recapitulate the pathophysiology of diseases (Xicoy et al., 2017). Therefore, results obtained with these cells require validation and comparison with more relevant experimental models. Primary cell lines are a more reliable cell type as they do not have a tumor origin or were not manipulated, and, therefore, more closely recapitulate the characteristics of neural cells *in vivo*.

#### Primary Cell Cultures

Primary cells represent the most used and reliable cell type for *in vitro* studies because they are similar to cells involved in the tissue response *in vivo* (Table 1). These cultures are not always the first choice due to experimental constraints, like ethics and economic issues. However, they are an excellent tool to study cell behavior prior to *in vivo* studies. CNS neural primary cells are obtained by dissociation of excised CNS tissue explants and subsequent isolation and plating. For *in vitro* studies, CNS primary cells are most commonly obtained from animal models like rat and mouse, as one has very limited access to human CNS biopsies. With the recent advances in neuronal cell derivation from (human) pluripotent stem cells, neuronal cultures derived from these cell sources are emerging as a powerful tool for *in vitro* modeling (Song et al., 2016; Chen W. et al., 2018).

*In vitro* primary neuronal cultures are widely employed in microelectrode research for the testing of new surface modified materials with improved biocompatibility and to investigate the effects of surface topographies in the enhancement of neuronal adhesion, neurite outgrowth and electrochemical performance (Chapman et al., 2016; Catt et al., 2017; Seyock et al., 2017; Zöndör and Thoumine, 2017).

The use of single isolated cell types, despite being useful for the investigation of specific biochemical and morphological responses once in contact with a surface, excludes the possibility to study cell interaction with the material in the presence of the glial and neuronal cells crosstalk. A common approach to improve the *in vitro* assays is the use of mixed glial cultures with neuronal cells, obtained in a single isolation procedure, as a strategy to mimic the environmental characteristics and cellular events involved in astrogliosis. The use of mixed glial cells allowed to increase the physiological relevance of *in vitro* testing and to monitor the neuroinflammatory process and microelectrode modifications under manageable and reproducible conditions. Mixed glial and neuronal cultures were used as an efficient *in vitro* glial scar model for the screening of new design coatings for microelectrodes (Achyuta et al., 2010; Sommakia et al., 2014) (see Table 1 for examples). However, despite useful and cost-effective if compared to *in vivo* studies, primary neural cell cultures also present limitations. The isolation procedures are challenging and require appropriate expertise (Uysal et al., 2018). In addition, primary cell lines do not divide (as in the case on neurons) or do not divide indefinitely as immortalized cell lines, hence the number of cells obtained for each isolation is substantially reduced, limiting the number of experiments and the amount of sample for molecular studies (Gordon et al., 2014). Besides the difficulty in their manipulation, primary neural cells, are isolated in early stages of development, therefore they can result unapt for the study of processes that are only observed in the adult or lead to unaccurate results, as some cellular responses can only be observed in early stages of development. The neural differentiation protocols, including for embryonic stem cells (ESCs) or induced pluripotente stem cells (iPSCs), are also laborious and expensive and can lead to different maturation properties (Verpelli et al., 2013; Engel et al., 2016).

Common to conventional immortalized and primary cell cultures conducted in 2D substrates, is the loss of the ECM composition and structure, cell-ECM and cell-cell interactions (namely, the neuronal network), and cell mechanics of the tissue of origin, which, inevitably, results in a different cell behavior compared to *in vivo* (Tekin et al., 2018). Thus, conventional cell cultures cannot provide detailed information about the interactions of electrode materials with the neural tissue in the initial phases of acute injury or the process of foreign body response in pathological environments. Whence, the necessity of developing complementary models in order to properly study the host reaction to microelectrodes.

#### 3D *in vitro* Cell Cultures

With progress in the field of tissue engineering, one is observing an increase in the number of reports of mixed culture systems conducted in 3D scaffolds (Table 1). The use of 3D matrices provides additional dynamics to the application of *in vitro* platforms for glial scar modeling, offering a valid and reproducible system to implement microelectrode research prior to *in vivo* studies. Several types of 3D scaffolds for neural cultures have been developed. These engineered scaffolds can be based on natural or synthetic materials (mostly polymers). These constitute a great improvement for *in vitro* studies in terms of increase in complexity and open the way to a vast window of applications in nervous system modeling (Ko and Frampton, 2016). Jeffery et al. (2014) developed a photocrosslinkable and tunable hyaluronic acid-based hydrogel scaffold for mixed glial cultures and high throughput screening of microelectrode materials. A commercially available synthetic polystyrene scaffold was shown to support neuronal cell growth and differentiation. It has been already tested for the development of a 3D model of neuroinflammation employing embryonic primary cortical neurons that are able to grow, interact and form networks possessing electrical activity in the presence of mixed glial cultures (Smith et al., 2015). We have proposed the use of a alginate-based simple and reproducible astrocyte 3D culture system that mimics many features of astrogliosis (Rocha et al., 2015). Using this platform, we established the ECM mechanical properties as a key modulator of astrogliosis. Spencer and coworkers developed a type-1 collagen gel with mixed primary embryonic neural cultures as an *in vitro* model of glial scar to investigate the effects of micromotion around neural implants (Spencer et al., 2017a). Koss et al. (2017) recently developed a hyaluronic acid-based 3D hydrogel model to study the process of glial scar formation in response to implantable microelectrodes. The biocompatibility of this system allows the encapsulation of primary oligodendrocytes, microglia and astrocytes and has shown to reproduce the typical features of the *in vivo* glial scar process.

The additional advantage of using 3D systems is the possibility to manipulate and tune scaffold composition through the incorporation of different matrix components and bioactive factors to promote cell survival, migration, and differentiation in a 3D context. The objective is to generate 3D structures with mechanical and structural properties as similar as possible to the ones of the CNS tissue (see Table 1 for examples). Despite the great advances in this field, these systems still present some constraints. Some biomaterials used for scaffold production are characterized by a high modulus compared to the neural tissue and can lead to altered cell viability, proliferation, and differentiation. Conversely, soft biomaterials are more difficult to handle. The design of matrices with a nanosized microstructure and topographical cues that fully mimic the one found in the nervous tissue was still not attained. The procedures of cell extraction for molecular analysis after testing, scaffold processing, and imaging become more challenging in 3D, limiting high-throughput studies. Additional complications are related to the cell culture conditions: 3D scaffolds can constitute a physical barrier that limits oxygen perfusion, nutrient supply and accumulation of toxic compounds that can cause cellular alterations or apoptosis. Optimizations are still required to ensure a versatile cell encapsulation for different cell types and controlled culture conditions as close as possible to the *in vivo* environment. This is even more challenging in the context of disease modeling, where cells must be induced to display specific pathological profiles.

It is expectable in the future that the continuous progresses attained in the tissue engineering field, particularly related to improved scaffolds/matrices development, will allow the development of better *in vitro* systems that recapitulate neural tissue architecture, in an effort to minimize the gap between *in vitro* and *in vivo* experiments.

### *Ex vivo* Models

The development of 3D *in vitro* models of brain tissue, as stated above, represents an attractive tool for researchers working in the neurosciences field and their use in microelectrode research would have a great impact on the screening of new biomaterials for biomedical applications. However, additional optimizations are still required to allow a consistent application in neuroscientific research. A potential alternative that enables researchers to get closer to *in vivo* conditions is the use of *ex vivo* excised brain/spinal cord tissues.

#### Organotypic Cultures

Tissue explants can be extracted from euthanized animals or obtained from human biopsies and cultured *in vitro*. The great advantage of organotypic cultures compared to artificial *in vitro* systems is the preservation of the native cytoarchitecture with the maintenance of intact neuronal networks. Although several types of *ex vivo* models have been described in literature (Mii et al., 2013; Nery et al., 2015; Jones et al., 2016; Neville et al., 2018), brain slice cultures from rodents are the most established and widely used as a system of election for neurophysiological investigations, neuropharmacology and as a model of disease. The procedure consists in the isolation of specific districts from the whole brain, their dissection in slices and incubation under controlled conditions. The possibility to obtain several slices from a single animal constitutes an additional advantage in terms of reduction of the number of animals for experiment and of related costs.

Brain slice cultures have been well established from different brain regions (Table 2). Two types of brain slices preparations exist: acute slices from the adult brain, with a short life and mainly used for electrophysiological recordings, and organotypic slices from neonatal animals. The latter are the most diffused *ex vivo* platforms for the study of many physiological and pathological conditions, thanks to the possibility to reproduce, by external intervention, the hallmarks of diseases that occur *in vivo* (Magalhães et al., 2018). The great success of this system is due to the simplicity of the procedure and manipulation by mechanical or pharmacological treatment, as well as the possibility to perform electrophysiological recordings on bioelectric activity. Based on these features, organotypic cultures can constitute an ideal model for the long-term assessment of the complex host reaction to microelectrodes or for high-throughput biocompatibility studies of new materials and surfaces. Nevertheless, few works can be found in the open literature (Kristensen et al., 2001; Huuskonen et al., 2005; Ereifej et al., 2013a; Usmani et al., 2016; Leclerc et al., 2017). A possible explanation for this fact could be related to the preferential use of *in vivo* models as the gold standard for microelectrode testing. Although necessary for the translation of new materials to the clinic, *in vivo* experiments have ethical issues, they are expensive, time-consuming and unapt for screening studies due to their complexity. The great advances achieved by *in vitro*/*ex vivo* systems can be the successful strategy to accelerate microelectrode research prior to *in vivo* testing. The controllable and reproducible conditions make them suitable to identify strategies to mitigate neuroinflammation, to prevent the early biochemical and corrosion-related events at the interface electrode-neural tissue and to impair foreign body response. Nevertheless, although organotypic cultures maintain the 3D cytoarchitecture, slice preparation causes an unavoidable axotomy of the brain tissue and neuronal death. This physical damage is accompanied by loss of blood flow, and consequently jeopardize oxygen perfusion and nutrient supply. As occurs in primary neural cultures, organotypic cultures are derived from animals in early stages of development and require extensive periods of culture for their maturation for use in post-developmental studies and assessment of pathophysiological processes. Another important caveat is the lack of the BBB and circulating immune cells. In the context of testing materials for neural devices, we previously showed that these factors play a crucial role in the process of foreign body response. BBB dysfunction and cell infiltration are also associated with several neuropathological processes. This can limit the physiological relevance of organotypic cultures for disease modeling (Humpel, 2016). Hence, researchers are developing innovative approaches combining microfluidic technologies with cellular vascular structures to mimic BBB microarchitecture and improve culture conditions for long term studies (Xu et al., 2016). These authors proposed a new and dynamic in vivo-like three-dimensional microfluidic system to replicate the BBB *in vivo*. Despite these limitations, organotypic cultures are still one of the most relevant models and can represent a fascinating tool to reduce the differences between *in vitro* and *in vivo* studies (some examples are present in Tables 1, 2).

**Table 2:** Organotypic cultures as a model of neurological and neurodegenerative diseases.

Disease	Type of organotypic slice	Induction of disease	References
Parkinson′s disease	Parasagittal nigrostriatal slices	Slices incubation with 6-hydroxydopamine (6-OHDA)	Kearns et al., 2006
	Organotypic midbrain slices	Transfection with truncatedα-synuclein (A53T)	Zach et al., 2007
	Nigrostriatal organotypic slices	Mechanical cutting of dopaminergic fibers from substantia nigra to striatum	Cavaliere et al., 2010
	Ventral mesencephalon organotypic slices	Unilateral microinjection of 6-hydroxydopamine (6-OHDA)	Stahl et al., 2011
	Sagittal nigrostriatal slices	mechanical transection of the medial forebrain bundle	Daviaud et al., 2014
	Coronal nigrostriatal slices	Injection of rotenone	Ullrich and Humpel, 2009
	Cerebellar nigrostriatal slices	Slices incubation with 1-methyl-4-phenylpyridinium (MPP+)	Chong et al., 2015
Epilepsy	Organotypic hippocampal slices	Slices incubation with Kainic acid	Järvelä et al., 2011; Jung et al., 2013
	Organotypic hippocampal slices	Slices incubation with kainic acid or n-methyl di-aspartate	Prasad Tripathi and Ayyannan, 2017
	Organotypic hippocampal slices	Slices incubation in a Neurobasal/B27 serum-free medium	Magalhães et al., 2018
Alzheimer disease	Organotypic hippocampal slices	P301S Alzheimer disease mouse model	Mewes et al., 2012
	Organotypic coronal brain slices	Co-transfection with amyloid precursor protein cDNA or human tau4R0N cDNA	Van Kanegan et al., 2016
	Organotypic hippocampal slices	APPsdl mouse model	Penazzi et al., 2017
	Organotypic hippocampal slices	3xTg-AD mouse model	Croft and Noble, 2018; Jang et al., 2018
Traumatic brain injury	Organotypic hippocampal slices	Focal mechanical trauma at the CA1 region	Schoeler et al., 2012; Krings et al., 2016
	Organotypic hippocampal slices	Tissue deformation by mechanical stretching	Choo et al., 2013; Lamprecht et al., 2017
Stroke	Organotypic coronal brain slices	Exposure to oxygen glucose deprivation	Wang et al., 2006;
	Organotypic hippocampal slices	Exposure to oxygen glucose deprivation	Hall et al., 2009
Spinal cord injury	Organotypic spinal cord slices	Exposure to hypoxic condition	Kim H.M. et al., 2010
	Organotypic spinal cord slices	Slices incubation with kainic acid	Mazzone and Nistri, 2014
	Organotypic spinal cord slices	Mechanical damage using weight drop model of injury	Labombarda et al., 2013; Pandamooz et al., 2019

*APPsdl, amyloid precursor protein gene mutation; A53T, point mutation human α-synuclein protein; CA1, “Cornu Ammonis” 1 hippocampal region; MPP+, (1-Methyl-4-phenylpyridin-1-ium); 6-OHDA, 6-hydroxidopamine [5-(2-aminoethyl)benzene-1,2,4-triol]; P301S, microtubule associated protein Tau (OMIM 157140 genetic mutation); tau4R0N, human microtubule associated protein tau, transcript variant 3, mRNA; 3xTg-AD, transgenic Alzheimer disease mouse model.*

Finally, as a duty of each and every scientist, the use of *in vitro*/*ex vivo* models must be encouraged in order to improve the ethical acceptability of research in the fulfillment of the principles of Replacement, Reduction, and Refinement (3R’s) (Lossi and Merighi, 2018).

### *In vivo* Models

Different types of *in vivo* studies have been carried out in microelectrode research to evaluate therapeutic efficacy, durability and safety of microelectrodes. In this type of studies rats and mice are the most common model of choice. The animal disease models that are used to assess the efficacy of neurostimulation therapies are several (Table 1). The main categories are represented by animal models of neurodegenerative disease such as Parkinson’s disease (Badstuebner et al., 2017; Musacchio et al., 2017), Alzheimer’s disease (Leplus et al., 2018), epilepsy (Desai et al., 2016), sensory-motor deficits due to spinal cord injury (Capogrosso et al., 2018), blindness (Tang et al., 2018), hearing loss conditions (Allitt et al., 2016) and ischemic models (Yang et al., 2017). Large animals such as cats, dogs, sheep, pigs, and non-human primates, are used for chronic studies on the efficacy and safety of neural stimulators. They concern the analysis of both biotic and abiotic reactions on the tissue-electrode interface in long-term experiments (Shepherd et al., 2018). The employment of large animals for these types of investigations is recommended because their anatomy perfectly mimics the environment in which microelectrodes will be applied, allowing the use of all the device components in their real size. Moreover, the full inflammatory component is present *in vivo* as opposed to *in vitro*/*ex vivo* models. The surgical procedure in *in vivo* experiments generally consists in the exposition of the skull in a deeply anesthetized animal, the production of one or more drills in the vicinity of the target region, the insertion of the neural implant in a specific site and fixation of the plugs with dental cement, followed by continuous monitoring to assess the recovery of the animal, integrity of device and/or efficacy of the therapy (Fluri et al., 2015). Besides the damage caused by electrode implantation, the majority of *in vivo* experiments are carried out by tethering the animal to an external component through cables, causing severe discomfort. To overcome this, new wireless microstimulation technologies were developed to ensure better freedom of movement, allowing the reduction of distress (Fluri et al., 2017; Pinnell et al., 2018). Besides the use of animal models of disease, chimeric mice models have found great utility for answering important biological questions concerning the role of different cell types in the process of foreign body response. Chimeras are animal models with two or more different genotypes experimentally obtained by transplanting cells or organs from another organism. Bone marrow chimeric mice were employed to investigate the contributions of different cells in the mechanism of foreign body response (Ravikumar et al., 2014). Sawyer et al. (2014) generated chimera mice between wild type and MCP-1 knock out mice, assessed the key-role of MCP-1 in the enhancement of neuronal loss and showed that its inhibition can be an effective strategy to prolong the lifetime of implantable microelectrodes. Bedell and co-workers recently developed chimeric mice lacking cluster of differentiation 14 (CD-14) genes in myelinating cells and blood-derived macrophages. They demonstrated that targeting CD-14 in blood-derived macrophages improved microelectrode performance in long term experiments (Bedell et al., 2018a). Genetically engineered animal models are another successful tool in microelectrode research. Mice lacking specific genes involved in neuroinflammation and immunity were employed to investigate the biochemical pathways involved in the foreign body response, the identification of pharmacological targets (Kozai et al., 2014; Bedell et al., 2018b; Hermann et al., 2018a,b) and material testing (Lee et al., 2017). Mice carrying cell specific fluorescent tags have been shown to provide great advantages in the study of the contribution of different cells in a single animal as well as a valuable alternative to immunostaining steps (Sunshine et al., 2018; Eles et al., 2019). Gaire and colleagues recently developed a quadruple-labeled mouse with specific fluorescent tags for oligodendrocytes, microglia, neurons, and astrocytes and investigate the process of nervous tissue response to “Michigan” array silicon microelectrodes (Gaire et al., 2018b).

Substantial improvements have been implemented in the quality and quantity of *in vivo* investigations: new bio-imaging tools were applied in microelectrode research to make *in vivo* works more explanatory. Two-photon laser scanning microscopy allows imaging of living animals with elevated resolution. It has been used to investigate the nervous tissue response to new electrode coatings (Eles et al., 2017), glial cells characterization (Wellman and Kozai, 2018) or live calcium imaging (Kondo et al., 2017). Optical coherence tomography is a minimally invasive technique that has been proposed to be used in combination with two-photon laser scanning microscopy to provide high resolution angiography of damaged tissues around microelectrodes (Hammer et al., 2016). As an alternative to laborious histological and staining procedures on sectioned brains, x-ray micro CT scanning has been proposed as a high-resolution, time and cost saving procedure that allows a 3D x-ray scanning of the entire brain to quantify and characterize the lesions caused by electrode implantation (Masís et al., 2018).

The growing scientific interest in neural interfaces in the last decades is confirmed by the multitude of *in vivo* works focused on the testing of new and fully biocompatible coatings (Du et al., 2017; Spencer et al., 2017b; Shen et al., 2018; Vitale et al., 2018), less invasive implantation strategies (Tawakol et al., 2016; Shoffstall et al., 2018b) and new designs with improved electrical performance (Ferlauto et al., 2018; Xu et al., 2018) and with longer durability.

Despite being considered the ultimate model to test microelectrode prior clinical tests, *in vivo* models present some important drawbacks as previously mentioned. An important aspect to consider is the elevated costs and time required for animal experiments, as well as the resulting ethical constraints. *In vivo* experiments are complex and demand adequate facilities and technical expertise. As discussed above, transgenic mice offer great advantages, however, they have an extremely high cost due to their production and maintenance. More importantly, the effects of such modifications can lead to altered phenotypes that depart from the real scenario. In the context of device testing, *in vivo* experiments are quite laborious and require long periods of time to assess the long-term performance of microelectrodes or the biological and behavioral effects of specific neurostimulation therapies. The complexity and invasiveness of the experimental techniques do not always enable scientists to identify the early biochemical and material-related events at the interface electrode-neural tissue and the strategies to mitigate neuroinflammation.

Despite these weaknesses, *in vivo* studies constitute the gold-standard for the investigations on neural implants, where the results obtained by previous *in vitro* testing find their effective validation. They represent the final step in the long process of microelectrode design and testing before the application in the clinic. A rigorous progression along all the steps determines the success of new technologies. This is even more important in the case of new materials or designs, where a careful preclinical assessment is necessary to minimize the risk of failure once applied to human patients.

## Future Perspectives

### Increasing Complexity

Preclinical studies are paramount in the development and testing of new materials for neural implants. As a consequence, the demand for more reliable *in vitro*/*ex vivo* models is growing to satisfy the need for assessing of the increasing number of new materials being proposed for this application, improve the quality of device testing and reduce the time between prototyping and commercialization of new products. As discussed in the previous section, several *in vitro* models and new platforms have been described in the literature (Table 1), but they still need to be explored, tested and eventually adapted for microelectrode research.

The use of *ex vivo* platforms from tissue explants can represent a valuable solution that fits perfectly these purposes. Brain organotypic cultures have been widely used in the last years as an excellent model for a great number of applications. They were employed to study physiological (Svensson and Chen, 2018), and pathological conditions (Tan et al., 2017) or for the screening of new therapeutics (Minami et al., 2017). Furthermore, several organotypic cultures have been established as models of neurological and neurodegenerative diseases (Table 2), for which neural implants have been proposed as therapeutic strategies. This places organotypic models that mimic a pathological environment in a privileged position to serve as platforms for microelectrode testing and a potential strategy to move closer to the *in vivo* scenario. Moreover, thanks to the preservation of intact neural circuitry, organotypic cultures are particularly suitable to perform electrophysiological studies, analyze microelectrode performance and assess astrogliosis.

Developments in the stem cell biology field have also contributed to the establishment of new *in vitro* models of human disease, aiming at an increase of complexity to reach the relevance of the *in vivo* environment, while maintaining the controllability and manageability of *in vitro* systems. Organoids, self-organized 3D tissue cultures derived from stem cells, are currently leading these technologies and have already been developed for the majority of human tissues, including the brain. More recently, the advances in hiPSC reprogramming techniques are also contributing to a better performance of organoid platforms in mimicking human disease and serve as testing platforms for personalized medicine (Perkhofer et al., 2018). Lancaster and Knoblich (2014) the preparation of cerebral organoids prepared from hiPSC. The described methodology allows cell aggregates cultured in Matrigel to mimic native brain tissue, originating different developing brain regions, namely, cerebral cortex, ventral telencephalon and retinal, among others, within 1 to 2 months. iPSC derived organoids represent an innovation in the field of *in vitro* disease modeling, offering a great opportunity to investigate pathophysiological mechanisms of neurological diseases with elevated reliability (Ho et al., 2018; LaMarca et al., 2018; Sun et al., 2018) and can also contribute to the field of implantable microelectrodes. In fact, Ormel et al. (2018) demonstrated that organoids can innately develop microglia and have a response to inflammatory stimuli that recapitulates neurons-glia interactions *in vivo*. This is an important aspect since glial cells, particularly microglia, are involved in a great variety of pathophysiological mechanisms.

**FIGURE 3:**
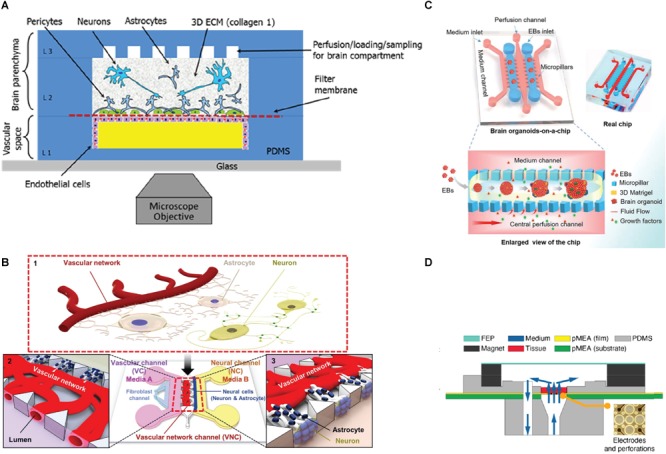
Schematic representations of 3D microfluidic systems. **(A)** Schematic view of a microfluidic device for 3D cell culture composed by a vascular channel (VC) for primary human brain-derived microvascular endothelial cells (hBMVEC), and a brain chamber for primary cell-derived human neurons, pericytes and astrocytes culture in a type I collagen matrix. Reprinted from Brown et al. (2015) with the permission of AIF publishing. **(B)** 3D microfluidic platform for the establishment of a neurovascular unit (NVU) including blood-brain barrier (BBB). The NVU is characterized by a VC composed by a co-culture of HUVEC and Primary human lung fibroblasts, and a secondary NC composed by a co-culture of neurons and astrocytes. Adapted with permission from Bang et al. (2017). **(C)** Organ-on-a-chip device for 3D culture and differentiation of brain organoids, showing an enlarged view of the component parts and a flow chart showing the development stages of hiPSCs-derived brain organoids (Wang Y. et al., 2018) published by the Royal Society of Chemistry. **(D)** Vertical cross-section view of a perforating multi-electrode array (MEA) integrated in a PDMS device for long-term culture, live imaging, recording and stimulation of brain tissues and 3D cultures (Killian et al., 2016).

Great advances *in vitro* modeling have been also achieved thanks to the application of microfluidics combined with 3D *in vitro* cultures (Rocha et al., 2016). Microfluidic platforms consist of polymer-based platforms for *in vitro* culture of cells that allow control and manipulation of microenvironment and fluids (see Figure 3 for relevant examples). The use of these microdevices brings *in vitro* models to a whole new level, thanks to the possibility of modifying spatial organization by isolating specific districts and simulate 3D tissue architecture of the native tissue. These systems allow the continuous control of external conditions, conferring an added value to *in vitro* technology, and giving the possibility to reproduce new biological features that are not possible to achieve with conventional culture systems (as discussed in section “Organotypic Cultures” for the case of the BBB). Wang and colleagues developed an organ-on-a-chip system for long-term culture of brain organoids under controlled conditions. Brain organoids were cultured on Matrigel scaffolds with a sided channel for the culture medium supply and a central perfusion channel, allowing a continuous culture medium flow and providing an improved proliferation and neural differentiation compared to static culture conditions (Wang Y. et al., 2018). Liu et al. (2018) combined multielectrode array technology with a microfluidic perfusion system for organotypic hippocampal slices as a platform for high throughput drug discovery. Microfluidic vascular models have been developed and applied to brain-on-a-chip platforms, enabling scientists to improve the quality of culture conditions and get even more close to *in vivo* dynamics (Osaki et al., 2018; Wang, 2018). New microfluidic devices that model the BBB were fabricated and tested on 2D and 3D cultures, showing that BBB integrity and permeability simulates *in vivo* characteristics (Chin and Goh, 2018). Adriani et al. (2017) developed a 3D neurovascular chip composed by a central hydrogel co-culture of rat primary neurons and astrocytes, and two lateral channels hosting human umbilical vein endothelial cells and human cerebral microvascular endothelial cells. Bang et al. (2017) developed a 3D microfluidic BBB platform with a vascular channel (VC) composed by a co-culture of human umbilical vein endothelial cells and primary human lung fibroblasts directly interfacing with a neural channel (NC) composed by a co-culture of primary rat neurons and astrocytes to simulate the neurovascular unit. They showed that this platform displayed permeability, cellular contacts and synaptic structures comparable to the *in vivo* BBB, suggesting its great potential for the drug screening for neurological diseases. Microfluidic technology can conduct *in vitro* culture system to a more complex and realistic level providing many advantages and details that cannot be extracted with conventional *in vivo* models, such as the easy manipulation, low cost, and the possibility to investigate more intimately key mechanisms of diseases. These characteristics perfectly fit with ideal biological platforms for the testing of microelectrode materials developed in the last decades. 3D microfluidic systems can eliminate some of the limitations of 3D *in vitro* technology, creating new high-fidelity throughput systems that can improve the testing performance and reduce the cost and time for pre-clinical assessment. An additional advantage is the possibility to induce specific pathological features by external treatment for long-term experiments, offering the possibility to investigate in advance microelectrode performance and nervous tissue response under disease conditions.

### Design Solutions and New Materials for the Improvement of Microelectrode Durability and Biocompatibility

Despite remarkable developments in implantable microelectrodes for neuroprosthetics and DBS, additional investigations are still required to address the biocompatibility and the long-term durability issues. A critical issue is reducing the physical stress, local inflammation and electrode degradation caused by the reaction between electrode and tissue interface while maintaining the electrical sensitivity of the electrode (Prodanov and Delbeke, 2016). To tackle these issues, multiple material-based strategies regarding this problem have been suggested, including (i) chemical modification of the electrode materials, (ii) new design of electrode structures, and (iii) non-invasive and wireless approach using functional nanoparticles.

Biological and non-biological electrode modifications, especially through the surface coating of substrates and electrode sites, are the most commonly used strategies to improve interfacial mechanical mismatch (Aregueta-Robles et al., 2014). Advances in fabrication approaches for integrating conductive polymers (Kim et al., 2018), shape-memory polymers (Shoffstall et al., 2018a), hydrogels (Crompton et al., 2007; Frampton et al., 2007) and carbon nanotubes (Baranauskas et al., 2011; Bareket-Keren and Hanein, 2012) onto complex electrode structures, provide not only a chronically stable neural interface, but also an improvement in the electrode performance. The reduced surface area combined with low impedance and sensitivity provided by such materials make them suitable for either stimulation and recording applications (Vitale et al., 2015; Du et al., 2017; Pancrazio et al., 2017; Wang et al., 2019).

The additional advantage is that the bioactive molecules can be attached to the coating surfaces to increase stimulating/recording sensitivity. Employing composite materials for electrodes and coatings has also emerged as a promising strategy for upgrading electrode functionalities and biocompatibility. Heo et al. (2016) have reported improved biocompatibility in polyimide-based microelectrodes by coating them with PEG hydrogels containing Poly lactic-glycol acid (PGLA) microspheres loaded with the anti-inflammatory drug. On the other hand, Zhou et al. (2013) proposed a carbon nanotube doped PEDOT composite coating material onto the Pt electrode. They showed that this coating makes the electrode more stable with enhanced charge transfer capacity and tissue-electrode interaction. While chemical modification of materials is still being suggested as an efficient way of protecting both electrode and brain tissue, the long-term stability issue caused by the degradation and delamination of coating materials still remains as the challenge that needs to be overcome (Green et al., 2008).

**FIGURE 4:**
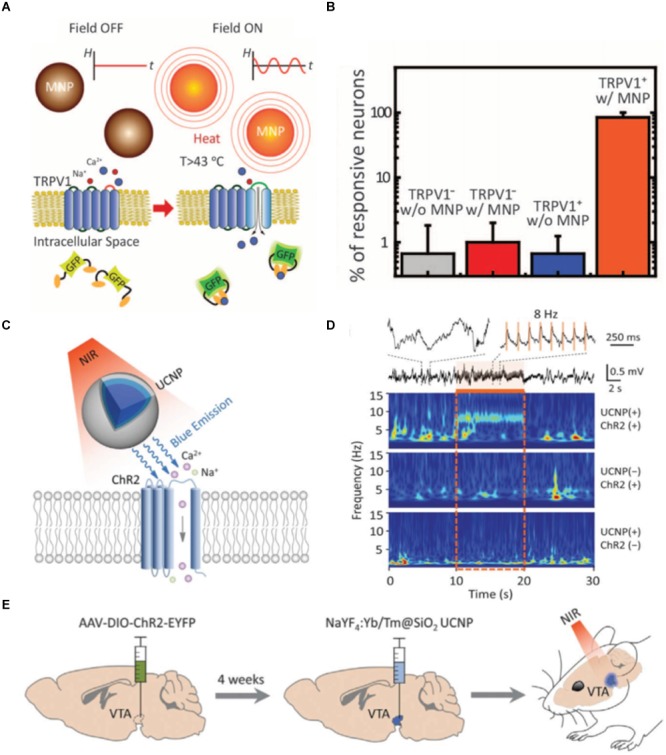
New strategies for deep brain stimulation using functional nanoparticles. **(A)** A schematic description of magnetothermal effect on transient receptor potential cation channel subfamily V member 1 (TRPV1) cells. **(B)** Comparison of the neuron reactivity under different conditions. Figures from Chen et al. (2015), reprinted with permission from AAAS. **(C)** A schematic description of nanoparticle-mediated near infrared (NIR) upconversion optogenetics. **(D)** Hippocampal local field potential response under NIR stimulation under different conditions. **(E)** *In vivo* experimental description of NIR stimulation of the ventral tegmental area of mice. Figures from Chen S. et al. (2018), reprinted with permission from AAAS.

Another attempt to reduce the immune response while enhancing functionality is to introduce new microelectrode designs. The development of the fabrication techniques of soft materials has enabled the production of ultrasoft and ultrathin electrodes with complex designs that minimize the mechanical mismatch of the electrode-tissue interface (Weltman et al., 2016). Recently, Kim et al. have fabricated ultrathin polyimide-based polymer electrodes covered by bioresorbable silk film. They successfully demonstrated the integration of the ultrathin electrodes with a complex structure by allowing the silk to be dissolved and resorbed. This procedure encouraged the spontaneous wrapping process driven by the capillary effect at the material-tissue interface, generating greatly improved biocompatibility (Kim D.H. et al., 2010). Carbon nanotube-based soft fiber microelectrodes have also proved to have low impedance and effective therapeutic stimulation along with single-neuronal-unit signal detectable resolution, owing to their high surface area and electrical conductivity (Vitale et al., 2015). Compared to the similar dimension and surface environment, ultra-soft and ultra-thin electrodes have a great potential to significantly reduce inflammatory tissue response in the long-term scale (Du et al., 2017). However, as mentioned earlier, the balance between flexibility/softness and the electrical performance should be carefully considered when designing these type of electrodes (Wellman et al., 2018b). Implementing functional nanoparticles are attracting increasing attention as a non-invasive and remotely controllable method. Chen et al. (2015) succeeded in utilizing the magnetothermal effect of nanoparticles for DBS (Figure 4A,B). They injected Fe_3_O_4_ magnetic nanoparticles in the ventral tegmental area of mice and exposed them to the external magnetic field. When magnetic nanoparticles are exposed to the AC magnetic field, stimulation of neurons at the targeted brain region was triggered by the dissipated heat from the magnetothermal effect. Wireless neural stimulation was successfully performed 1 month after injection. Moreover, lower glial activation, less macrophage accumulation and neuronal loss have been reported compared to a stainless steel implant. Chen S. et al. (2018) recently proposed optogenetic treatment by shining near infrared light to molecular tailored upconversion nanoparticles (Figure 4C–E). They injected nanoparticles into the ventral tegmental area of the brain to stimulate deep neurons and successfully demonstrated that light treatment on upconversion nanoparticles can induce dopamine release from dopaminergic neurons, activation of inhibitory neurons, inhibition of hippocampal excitatory cells, and memory recall. Magnetoelectric nanoparticles are another great candidate for neural stimulation. It has been already proven that the piezoelectric materials can generate electric signals under the acoustic wave and can induce neural cell differentiation (Chen et al., 2019). As ferromagnetism and ferroelectricity are coupled to each other, applied external magnetic field can induce variation in electric polarization of nanoparticles, causing a change of the electronic structure at the particle surface and therefore facilitating stimulation of the tissue deep inside the brain (Kargol et al., 2012; Guduru et al., 2015). As a non-invasive stimulating method, implementing magnetoelectric material is attracting significant attention. While these approaches using nanoparticles have great potential, biocompatibility and cellular uptake of these functional particles still remain as a problem to be solved (Adjei et al., 2014; Behzadi et al., 2017).

## Concluding Remarks

The field of the brain-machine interface is exponentially growing and comprises an important source of progress in many aspects of neurosciences. The application of bionic systems, neural prosthetics and neurostimulation for restoring/treating severe neuro-debilitating conditions and neurological diseases has attracted the interest of many researchers and clinicians. All these technologies require the use of implantable microelectrodes to interface with the CNS. They represent an essential tool that serves as a link between the electronic components and the neuronal networks, in order to ensure a stable electrochemical communication over time. Great success has been achieved by the clinical application of neural prosthetics in the improvement of the quality of life of patients that suffer from sensory-motor deficits. DBS has become a treatment of choice for movement disorders and neuropsychiatric diseases and is proving to be a relevant alternative for a multitude of other pathological conditions.

The recent progress in microfabrication techniques made possible the development of microelectrodes capable of simultaneous recording and stimulation with improved cell selectivity and spatial resolution. Despite the improvements in device fabrication, biocompatibility and electrochemical performance for long-term applications, unfavorable nervous tissue response and microelectrode failure are still significant limitations. The process of nervous tissue response to microelectrodes has been described by an acute and chronic phase. The acute phase represents the most critical step characterized by a series of pathological reactions triggered by BBB dysfunction and glial activation. The persistence of this neuroinflammation is responsible for the immune response and the formation of the glial scar in the chronic phase, which may lead to microelectrode failure. A better understanding of the signaling pathways involved in the acute and chronic responses is required in order to develop new design strategies to mitigate neuroinflammation and promote a successful integration. Several surface modified microelectrodes have been designed to provide minimal damage and establish a minimally reactive interaction with the brain tissue. However, additional studies are necessary to comprehend some of the key-cellular mechanisms implicated in the process of the glial scar formation around microelectrodes that remain to be elucidated. An important aspect in which research must be focused on is the binary role of the glial scar: several studies report a neuroprotective function of the glial scar in many pathological conditions, and its modulation has been suggested as a therapeutic approach to improve neuronal recovery and tissue regeneration. In the case of chronically implanted microelectrodes, the participation of the various glial subtypes to the nervous tissue response and how their activation states can be influenced to soften tissue damage and avoid rejection are still unclear aspects. Toward this end, the use of appropriate experimental models can provide significant advantages in the development and testing of biocompatible and durable neural devices.

In this review, we provide an overview of the current and potential experimental *in vitro, ex vivo*, and *in vivo* models to investigate the mechanisms of foreign body response to implantable microelectrodes. The progress in 3D tissue engineering and disease modeling opened the way toward the development of *in vitro* biological platforms with increased complexity and physiological relevance to be used for high-throughput studies before moving to *in vivo* animals. Organotypic culture systems are widely established *ex vivo* platforms which offer the possibility to simulate several pathological conditions and to isolate specific cerebral regions, ensuring the preservation of tissue architecture and synaptic organization for electrophysiological studies. While the use of organotypic culture systems as screening platforms for novel microelectrodes is still limited, their application is expected to grow in the near future, not only for the reasons mentioned earlier but also because these systems can contribute to significantly minimize the use of animal models. Additional implementations have been also achieved *in vivo* studies. Despite being considered the gold-standard for microelectrode safety and efficacy studies, the principal limitation of *in vivo* experiments is the difficulty to monitor tissue response in the initial phases of injury. New advanced neuroimaging techniques open a new window of opportunities to improve the relevance of *in vivo* assessment thanks to the possibility to study biochemical processes, cell behavior and structural modifications in real time with elevated resolution. The advent of iPSC technology has enabled to simulate more closely the pathophysiological cues that occur in human diseases, offering the relevant advantage to recapitulate molecular and biological characteristics of the human brain. Success is also being achieved by the use of microfluidic systems combined with 3D cell cultures and/or iPSC-derived organoids, which allowed for integrating mechanical and physiological dynamics to simulate organ-like functions and responses. They represent a cost-effective compromise between the versatility of *in vitro* models and physiological relevance of *in vivo* models, offering the possibility to model pathophysiological cues under simulated conditions.

In conclusion, researchers have now a great variety of relevant models that can be adopted to improve microelectrode research in all the phases of development and to address the scientific unknowns related to the nervous tissue response to microelectrodes. Ultimately, with further improvements of these *in vitro* models, one can expect the creation of optimal milieus, which can substantially replace animal experimentation for large scale studies.

## Author Contributions

All authors listed have made a substantial, direct and intellectual contribution to the work, and approved it for publication.

## Conflict of Interest Statement

The authors declare that the research was conducted in the absence of any commercial or financial relationships that could be construed as a potential conflict of interest.
